# New Insights and Methods in the Approach to Thalassemia Major: The Lesson From the Case of Adrenal Insufficiency

**DOI:** 10.3389/fmolb.2019.00162

**Published:** 2020-01-29

**Authors:** Maurizio Poggi, Irene Samperi, Lorenza Mattia, Arianna Di Rocco, Cristina Iorio, Salvatore Monti, Giuseppe Pugliese, Vincenzo Toscano

**Affiliations:** ^1^Department of Clinical and Molecular Medicine, “La Sapienza” University, Rome, Italy; ^2^Endocrine-Metabolic Unit, Sant'Andrea University Hospital, Rome, Italy; ^3^Department of Public Health and Infectious Disease, “Sapienza” University of Rome, Rome, Italy

**Keywords:** Thalassemia Major, adrenal insufficiency, synachten, endocrine comorbidities, cortisol peak

## Abstract

**Background:** Thalassemia Major (TM) is a complex pathology that needs a highly skilled approach. Endocrine comorbidities are nowadays the most important complications, including hypogonadism, hypothyroidism, diabetes mellitus, and bone diseases. Recent works stated that there could be a relevant prevalence of adrenal insufficiency (AI) present in TM, and this fact may become crucial, especially in case of major stressful events.

**Aim:** Test the reliability of the standard test to diagnose AI in a group of TM and correlate it with clinical, hematological, and radiological data.

**Methods:** We evaluated endocrine damages and the efficacy of iron chelation therapy in 102 patients affected by TM. AI was assessed by tetracosactide (Synacthen) 1 mcg iv (low-dose test, LDT) stimulation test. Patients with a subnormal response (peak cortisol < 500 nmol/L) were followed up to 5 years to check the symptoms and signs of AI.

**Results:** We found AI in 13.7% of the population studied. We did not find any correlation between AI and all data evaluated. Only female gender seems to be a protective factor. A follow up of the patients affected by AI showed no signs of adrenal crisis, in spite of no replacement therapy.

**Conclusions:** Our study shows a relevant prevalence of AI in TM, especially in males. The absence of an adrenal crisis, in spite of no replacement therapy, during the long-term follow up, seems to underline that current methods to evaluate AI, in TM, should consider a different and specific diagnostic test or different cut off for diagnosis.

## Introduction

β-Thalassemia Major (TM) is a hereditary disease characterized by an impaired production of hemoglobin chains which can be due to over 200 mutations of the β chains gene. These mutations induce an abnormal production of the same hemoglobin and cause a severe haemolytic anemia (Rund and Rachmilewitz, 2005). The main therapy of the disease consists of a regular blood transfusion regimen that, frequently, hesitates in iron deposition and excessive storage. The possibility of an adequate chelation therapy has dramatically improved the quality and the life expectancy of these patients (Borgna-Pignatti et al., 2005). Nowadays, these patients have the chance to survive until adult age, resulting in a clinical picture of this disease that is deeply different in comparison with the past (Angelucci et al., 2008; Pinto et al., 2019).

The iron deposition, related to a transfusion treatment, causes a cytotoxic effect in many organs between which are the endocrine glands. Endocrine complications represent one of the most relevant problems and, specifically, alterations of pituitary, thyroid, pancreatic and bone status are the most important (De Sanctis et al., 2018; Pinto et al., 2019). Less is known about the chance of suffering from adrenal impairment (AI). Since the pituitary-adrenal axis is necessary to face stresses, evaluation of adrenal function in all adult TM patients could be crucial.

Previous studies in children and adolescents affected by TM have reported variable prevalence of endocrine impaired functions (De Sanctis et al., 1995, 2011; Scacchi et al., 2010). Results regarding adrenal impairment are heterogeneous in terms of methods used, incidence and population studied (adults, adolescents, and children; Nakavachara and Viprakasit, 2013; Ambrogio et al., 2018).

In order to clarify the incidence of AI in TM patients, we decided to assess the pituitary/adrenal axis in a large group of adult patients, focusing not only on hematological and hormonal data but also on clinical history, thanks to a long follow up evaluation (up to 5 years).

## Materials and Methods

This study was performed at our Endocrine Unit dedicated to patients affected by hemoglobinopathies. We collected data from 102 adult polytransfused β-Thalassemic Major patients, 19–50 years old, including both males and females (47 and 55, respectively). All of them were regularly blood transfused and were treated with chelation therapy. Patients who were taking glucocorticoids or other drugs known to affect adrenal function were excluded. This is a retrospective analysis of patient records of routine care. Therefore, ethical review and approval was not required for the study on human participants in accordance with the local legislation and institutional requirements. The patients/participants provided their written informed consent to participate in this study. Assessment included general data, medical history collection, physical examination, blood samples at baseline and after stimulation test and T2^*^ sequences magnetic resonance imaging.

We collected personal data like age and gender. Clinical evaluation included height, weight, and blood pressure. In all patients, we evaluated the presence of other endocrine damage, in particular, the presence of hypogonadism, hypothyroidism, diabetes mellitus, hypoparathyroidism and bone disease, and evaluated serological positivity to HBV, HCV, and HIV. The adequacy of chelation treatment through medium serum ferritin levels and by liver T2^*^ magnetic resonance imaging (T2^*^MRI) was investigated. Finally, we collected hematological data to evaluate liver function and electrolyte serum levels. At baseline, blood samples for biochemical (ferritin, hemoglobin, sodium, potassium, hepatic transaminases, albumin) and hormonal assay (adrenocorticotropic hormone—ACTH, thyroid stimulating hormone—TSH, free thyroxine—fT4, dehydroepiandrosterone sulfate—DHEAS) were collected fasting, between 8:00 and 9:00 a.m. Soon after baseline, blood samples of all patients underwent a 1 μg cosyntropin test. Blood samples for total cortisol measurements were collected at baseline and at 30 and 60 min after iv injection of 1 μg cosyntropin. One vial of 0.25 mg cosyntropin (ACTH_1−24_, tetracosactide) was diluted in sterile normal saline solution to a concentration of 5 μg/ml. One microgram cosyntropin (0,2 ml) was injected through a short iv catheter (Cross et al., 2018).

Patients with a subnormal response of cortisol (cortisol post-dose < 500 nmol/L, in according to most recent recommendation) were strictly followed up for a period of 5 years to check clinical and hematological signs of adrenal failure, in order to promptly start a replacement therapy. The check included clinical evaluation such as blood pressure measurement and the research of other signs and symptoms of adrenal insufficiency, hematochemical, and hormonal evaluation.

All hormones were analyzed using a chemiluminescence immunoassay (CLIA). Sensitivity of assays was 0.25 pmol/L for ACTH and 0.5 nmol/L for serum cortisol; intra-assay and inter-assay coefficient of variations were 4.9 and 8.9% for ACTH and 4.3 and 5.5% for serum cortisol (LIASON XL Analyzer).

### Statistical Analysis

A descriptive analysis of all the sample parameters collected was carried out. The normality of the distribution of the continuous quantitative variables was evaluated through the Shapiro-Wilk test; the variables with Gaussian distribution were reported as mean and standard deviation (SD), while the variables with Non-Gaussian distribution were reported as median and interquartile range (IQR). The qualitative variables were presented as absolute frequencies and percentages. Odds ratios (ORs) and 95% confidence intervals (CIs) were calculated using logistic regression analysis; univariate and multi-variable analyses were carried out to evaluate the role of variables as risk factors determining the response of cortisol. A *p* < 0.05 was considered as statistically significant and all tests were two-sided. All statistical analyses were performed with the software R version 3.5.1.

## Results

A total of 102 polytransfused β-TM adult patients without previous diagnosis of adrenal insufficiency were identified. Herein we present all the data we collected the day the LDT was performed. Demographic, biochemical and hormonal data are reported in Table 1. Forty-seven patients (46.1%) were male, 55 were female with median age of 37 years old (range 19–50 years old).

**Table 1:** Patient's characteristics.

Sex	47M/55 F (46.1% male)^*^
Age	37 years (19–50)^*^
BMI	22.6 Kg/m^2^ (15.5–34.5)^*^
Hemoglobin	10.5 g/dl (8–12.3)^*^
Ferritin	42.2 ng/ml (7.5–492.7)^*^
^¥^Liver T2^*^ MRI	14 ms (0.9–46)^*^
Albumin	4.1 g/dl (3.4–5.2)^*^
AST	29 U/L (8–253)^*^
ALT	33 U/L (7–261)^*^
Sodium	139 mmol/l (129–144)^*^
Potassium	4.4 mEq/l (3.6–8.8)^*^
PAS	110 mmHg (80–135)^*^
PAD	70 mmHg (40–90)^*^
DHEAS	81.6 μg/dl (3.7–487.7)^*^
ACTH	20.2 pg/ml (5–144)^*^
HCV positivity	75.49%
HBV positivity	16.67%
HIV positivity	2.94%
**Endocrinopathies**	
Hypoparathyroidism	4.9%
Osteoporosis	65.69%
Diabetes mellitus	11.76%
Hypothyroidism	45.1%
Growth hormone deficiency	15.67%
Hypogonadism	64.71%

* All values are median. In brackets are expressed range values.

¥ *Available on 68 patients*.

*BMI, body mass index; ALT, Alanine transaminase; AST, Aspartate transaminase; DHEAS, Dehydroepiandrosterone sulfate; ACTH, Adrenocorticotropic hormone; HCV, hepatitis C virus; HBV, hepatitis B virus; HIV, human immunodeficiency virus; MRI, magnetic resonance imaging*.

Blood pressure (BP) was in the normal range with median systolic BP 110 mmHg (range 80–135 mmHg) and median diastolic BP 70 mmHg (range 40–90 mmHg). Fasting glycaemia was normal in the majority of patients (median values of 83 mg/dl; range 41–270). Liver function tests showed median alanine transaminase of 33 U/L (range 7–261) and median aspartate transaminase of 29 U/L (range 8–253), also albumin levels were normal (median 4.1 g/dl, range 3.4–5.2). Only 19 patients were serological negative for HBV, HCV, and HIV, for the remaining, 77 patients (75.49%) were HCV positive, 17 patients (16.67%) were HBV positive and 3 (2.94%) were HIV positive.

The entire group showed good levels of ferritin (median 42.2 ng/ml, interquartile range 24.7–88.5, range 7.5–492.7) and T2^*^ MRI (median 14 ms, range 0.9–46), signs of the efficacy transfusion and chelation therapy, as expected in a population treated in an Italian tertiary dedicated center.

Despite these results, we observed a high prevalence of global endocrine damage. In particular, our population showed 64.7% of hypogonadism, 45.1% of hypothyroidism, 11.7% of diabetes mellitus, 15.6% of growth hormone deficiency, 4.9% of hypoparathyroidism, and 65.6% of osteoporosis. The univariate logistic regression didn't show any correlation between adrenal insufficiency and any other endocrinopathy.

**Figure 1:**
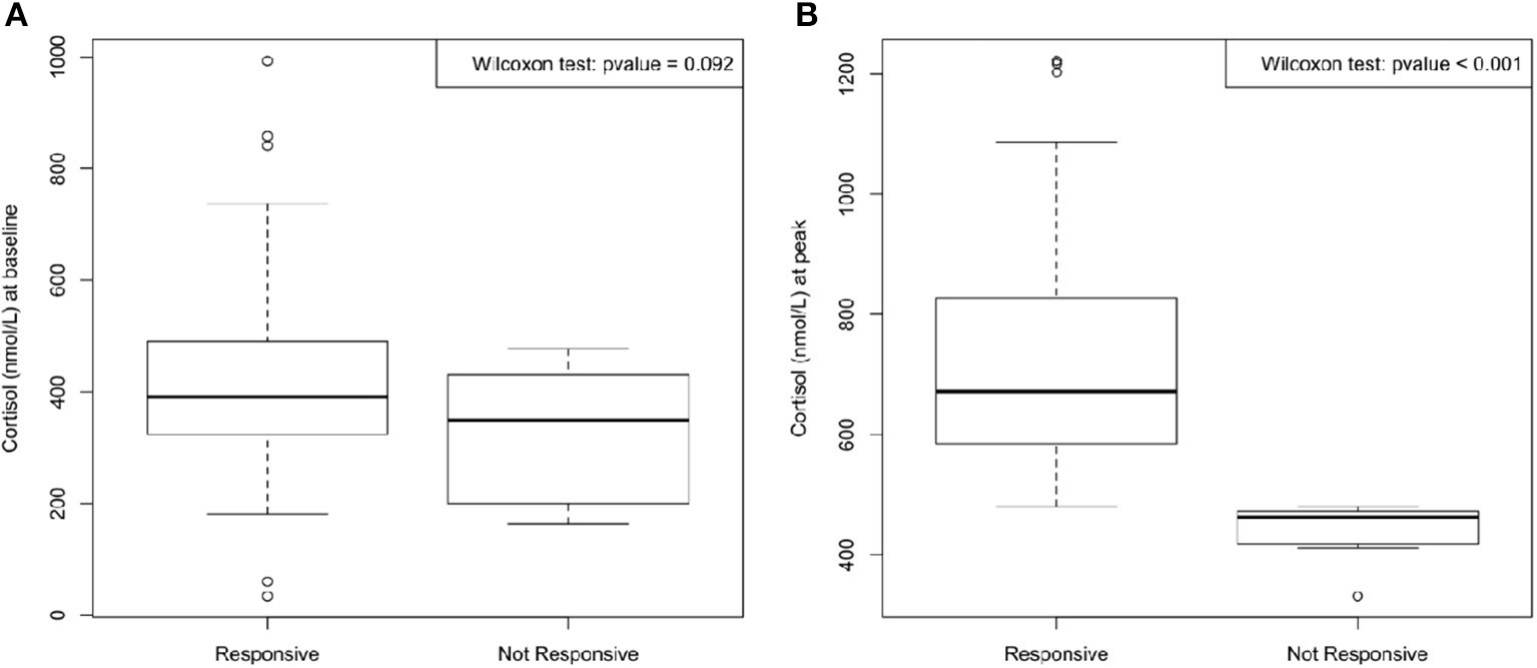
Median cortisol at baseline **(A)** and at peak (time 30 and 60 min) after 1 μg Cosyntropin test **(B)**. No significant difference between cortisol at baseline in responsive and in not responsive group was found; on the contrary, cortisol at peak reached a significant difference (*p* < 0.001) between the two groups.

Regarding the assessment of adrenal function, the main target of our work, 14 patients (13.7% of population) failed to reach the value of 500 nmol/L on LDT. Moreover, median cortisol peak value in the 14 patients with adrenal impairment (peak < 500 nmol/L) was 461 nmol/L (range 372–480 nmol/L) Figure 1.

When considering the failure to pass the LDT, the only significant factor was gender, in particular female gender resulted to be a protective factor against the failure to the LDT (OR 0.110; IC 0.023–0.522; *p*: 0.005).

Any statistical correlation was found with data of efficacy of chelation therapy (ferritin and T2^*^MRI) neither regarding liver function, assessed by transaminases and albumin serum levels.

None of the patients who did not achieve the cut-off during the LDT complains of any symptom potentially related with adrenal insufficiency. Therefore, we decided not to start daily replacement steroid treatment but to advice the treatment only when needed (in case of major stress).

In the follow up period of up to 5 years, nobody experienced any AI symptoms nor adrenal crises. Moreover, none of these patients needed to start steroid replacement therapy also in a more stressed clinical context such as fever, or intercurrent illnesses.

## Discussion

Nowadays patients affected by TM live longer thanks to better transfusion regimens and more efficient chelation therapies. This is the reason why physicians, especially endocrinologists, need to be faced with problems never experienced before. It is also important if we consider the high economic impact of the endocrine therapies (Ehrnborg et al., 2000). Over the past decade we have tried to extensively study different aspects of endocrine comorbidities (Poggi et al., 2010, 2011, 2016) but still much has to be done in this field. Endocrinopathies are one of the most important and challenging problems for the disease team involved in the cure approach. Studies performed in high skilled centers showed that endocrine failure could reach up to 60–70% for hypogonadism and bone disease and up to 30–40% for thyroid disease and diabetes mellitus. All these endocrine comorbidities considerably impair quality of life (De Sanctis et al., 2018).

Between endocrinopathies, it is crucial to diagnose adrenal impairment in this clinical setting, especially if we consider the possibility for these patients to be faced with acute stress or intercurrent illness. Moreover, TM patients frequently need splenectomy which is consider a major surgery that could precipitate adrenal function and hesitate in a fatal event, also because of an increased risk of post-splenectomy sepsis and thromboembolic events (Galanello and Origa, 2010). In accordance with this possibility, Matin et al. recommend adrenal function test prior to surgery (Matin et al., 2015).

Another relevant aspect is to consider the possibility of misdiagnosed AI in this clinical setting because of the overlap between typical symptoms of patients affected by anemia (Cascio and DeLoughery, 2017) and the non-specific pivotal signs and symptoms of adrenal failure, such as asthenia, fatigue, hypotension, abdominal pain (Borgna-Pignatti and Gamberini, 2011).

For all these considerations, we retain it is crucial that adequate and well-defined studies are conducted in order to fully evaluate and focus adrenal status in TM.

Recently several other authors published studies regarding the impairment of adrenal function in Thalassaemic patients (Poomthavorn et al., 2010; Scacchi et al., 2010; Uçar et al., 2016; Ambrogio et al., 2018). These studies are heterogeneous in term of population (TM or intermedia, children or adult, number of patients involved) and methods (insulin tolerance test, ACTH test with 1 mcg or 250 mcg, salivary cortisol) with different outcome in term of AI prevalence.

To explore this aspect, we conducted a retrospective study to define the real prevalence of AI in our population of TM.

To our knowledge, this study is the largest adult TM patients series tested for adrenal function to date. Moreover, differently from other authors (Matin et al., 2015; Ambrogio et al., 2018), we selected only patients affected by major thalassemia and excluded those affected by thalassemia intermedia and we evaluated only adult patient. All patients were followed by the same investigator (M.P.) during the follow up time.

To diagnose AI, we focused not only on biochemical data and on the LDT results but also on the real clinical significance of these data following the entire group in a long-term follow up to 5 years. Interestingly, despite the fact that 14 patients did not normally achieve the cut off during the SST, similar to data available in literature, none of our patients required steroid treatment. Moreover, we could not find any correlation with other factors, except with gender. This difference could be due to the different population studied (number of patient, median age, history of iron overload).

The initial result of our work, like others authors (Ambrogio et al., 2018; De Sanctis et al., 2018), was that biochemical adrenal impairment is not a negligible event which affects 14 patient (13.7% of our population). As suggested by the most recent consensus and guidelines (Husebye et al., 2014; Fleseriu et al., 2016), we used the cortisol cut-off of 500 nmol/L during LDT to determine normal adrenal function. In our cohort, patients who did not achieve this cut-off had median cortisol peak of 461 nmol/L (range 372–480 nmol/L).

Despite the fact that the insulin intolerance test is the gold standard in this setting, SST is widely used (Cross et al., 2018) with good accuracy, but probably with the need to adopt new and different cut off also in the general population (Karaca et al., 2011; Simsek et al., 2015; Burgos et al., 2019).

In agreement with this, Cho et al. (2014) tried to re-assess the cut-off for the different test (ITT, high and low dose SST). In their population they identify the threshold of 16 μg/dl (≃441.4 nmol/l) for the LDT: despite this result they underline the need to individualize the cut-off on the basis of the tested population.

Some authors described the presence of a chronic hyperactivation of the hypothalamic-pituitary-adrenal (HPA) axis in thalassaemic subjects, probably due to physical stress such as anemia or oxidative stress (Poomthavorn et al., 2010). They speculated the possibility that the 1-mcg-Cosytropin test could be a too low stimulus in an hyperfunctioning axis and it could not be a suitable diagnostic test for this condition.

The necessity to identify different, new and more stringent cut-off could be even more crucial in some clinical settings like thalassemia and hemoglobinopathies. Moreover, considering the clinical complexity of the disease and the presence of different comorbidities we need to focus and to avoid starting unnecessary steroid therapy. This is particularly important if we consider the impact that such therapy could have especially on bone disease.

Looking for differences between patients affected by lower cortisol peak and those with a good response to LDT, we noted that only female gender reaches a significant difference between subgroups and it could be considered a protective factor, as underlined recently by other groups (Ambrogio et al., 2018).

Notably, we did not find any correlation between cortisol peak and the major parameters of efficacy of chelating therapy (ferritin and T2^*^MRI) which were previously identified as predictive factors of endocrine impairment progression (Chirico et al., 2015). The lack of this correlation agrees to our previous work regarding other endocrine damage (Poggi et al., 2010). We could speculate that iron deposition is not the only actor in the induction and progression of endocrine damage but other factors such as chronic hypoxia or different tissue sensibility to iron overload must be taken into consideration. In agreement with other authors (Singh et al., 2018), we underline the importance of checking liver function to fully understand the LDT results. Nevertheless, we did not find any correlation between liver function parameters and LDT adequate responders. We can speculate that other factors as low serum protein, but not albumin which was in the normal range in our cohort, could alter the response to LDT. The other relevant aspect that we considered, which was never studied in the past, is the long term follow up (up to 5 year) that allows us to shed light on this endocrine complication.

During the follow up period we never collected any medical data that could be related to a progressive or an acute failure of adrenal function (even during major stressful clinical situations like fever or surgery). This result could suggest that the impairment of adrenal disease, as stated by the result to dynamical hormonal evaluation, is not a clear clinical entity but only a laboratory data, probably related to the fact that, in the clinical context of thalassemia, we cannot adopt the same tools as in different condition of hypoadrenalism.

Our experience, for first time to our knowledge, shows the limit of the current methods to investigate adrenal insufficiency in adult TM. Once more, the lesson from the case of adrenal insufficiency, as the title of this paper states, remind us how of the importance of using specific and accurate diagnostic methods for different clinical conditions and this could be particularly true in a very complex pathology like TM that needs dedicated and highly skilled resources. We think that by only using specific, accurate and validated methods can we do a real diagnosis.

In conclusion, we have reported a large series of 102 adult TM patients on adequate transfusion and chelation treatment who underwent LDT to assess adrenal function. Fourteen patients (13.7%) did not achieve the threshold of cortisol value of 500 nmol/L, which is considered a normal response to the test. Female gender is the only statistically significant parameter being associated to normal response to LDT. None of our patients presented signs or symptoms of adrenal insufficiency at the time of the test and during the long-term follow up. We speculate that the threshold of 500 nmol/L is not adequate for this population and a lower limit should be used. In this work, the median cortisol peak of non-responders was 461 nmol/L, therefore we suggest that a lower value should be adopted, considering the absence of suggestive clinical signs of AI reported among these cases, but more studies are needed to support this hypothesis.

## Data Availability Statement

The datasets generated for this study are available on request to the corresponding author.

## Ethics Statement

Ethical review and approval was not required for the study on human participants in accordance with the local legislation and institutional requirements. The patients/participants provided their written informed consent to participate in this study.

## Author Contributions

MP, IS, LM, CI, SM, GP, and VT: conceptualization, investigation, methodology, and writing-original draft. AD: data curation and formal analysis. MP: project administration, supervision, and validation.

### Conflict of Interest

The authors declare that the research was conducted in the absence of any commercial or financial relationships that could be construed as a potential conflict of interest.
